# Cardiac Metastases from Choroidal Melanoma

**DOI:** 10.1002/ccr3.6080

**Published:** 2022-07-18

**Authors:** Ammar Madani, Nabil E. Omar, Ghulam Mustafa, Mahir Petkar, Samah Mohamed, Maryam Al kuwari, Sabir Abdul Karim, Reyad Mohsen

**Affiliations:** ^1^ Department of Medical Oncology, National Centre for Cancer Care and Research Hamad Medical Corporation Doha Qatar; ^2^ Pharmacy Department, National Centre for Cancer Care and Research Hamad Medical Corporation Doha Qatar; ^3^ Department of Nuclear Medicine, National Centre for Cancer Care and Research Hamad Medical Corporation Doha Qatar; ^4^ Department of Pathology Hamad Medical Corporation Doha Qatar; ^5^ Department of Radiology Hamad Medical Corporation Doha Qatar; ^6^ Department of Cardiology Hamad Medical Corporation Doha Qatar

**Keywords:** heart, melanoma, choroid, uvea, ocular

## Abstract

In patients with uveal melanoma, cardiac metastases can present without any symptoms. It is becoming more common than previously thought and highlights the importance of routine surveillance after definitive treatment.

## Graphical abstract

Uveal melanoma can recur with cardiac metastases which are often asymptomatic. This highlights the need for routine surveillance by CT or PET CT every 6–12 months.

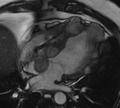

## 1 INTRODUCTION

The most common primary intraocular malignancy is uveal melanoma. The peak incidence of occurrence is from 55 to 65 years.^1^ Annually around 6 million people are diagnosed with ocular melanoma.^2^ It predominantly arises in the uvea and differs from cutaneous melanoma by several features like high proportion of liver metastases, poor response to systemic treatment, and late appearance of metastases.^3^ Uveal melanoma originates from melanocytes in iris, choroid, or ciliary body of the eye. The common sites of metastases are liver, lung, and bone. Other sites can include skin, kidney, and brain. Death usually results from distant spread to the liver.^4^, ^5^ Even though the metastases from ocular melanoma is less frequent compared with cutaneous, but when it does occur, it is invariably fatal resulting in death within 1 year of start of symptoms.^6^

Although the liver is the most common site for metastases from uveal melanoma, the heart is another site that is underdiagnosed.^7^ The patient remains clinically free of cardiac symptoms in majority of cases when there is metastasis to heart and symptoms related to other visceral metastases may be more prominent.^8^

Here, we report this case in view of diagnosis of uveal melanoma at a young age, delayed and extensive systemic recurrence without the presence of any clinical symptoms.

## 2 CASE PRESENTATION

A 56‐year‐old Egyptian male, known case of gouty arthritis on allopurinol initially presented in mid of 2014 with progressive visual loss, later he was diagnosed with left eye choroidal melanoma in his home country, he underwent left eye enucleation in Egypt, but histopathology report was not available. Staging work up by CT scan of the chest and abdomen was negative. He had an inferior wall myocardial infarction in April 2016 and underwent primary percutaneous coronary angioplasty with stenting to his right coronary artery (RCA). He also had residual 30% disease in the left anterior descending artery. The echocardiogram during the time was normal with a left ventricular ejection fraction of 50%–55%. Repeat coronary angiography in April 2018 showed patent stent in proximal RCA.

**FIGURE 1:**
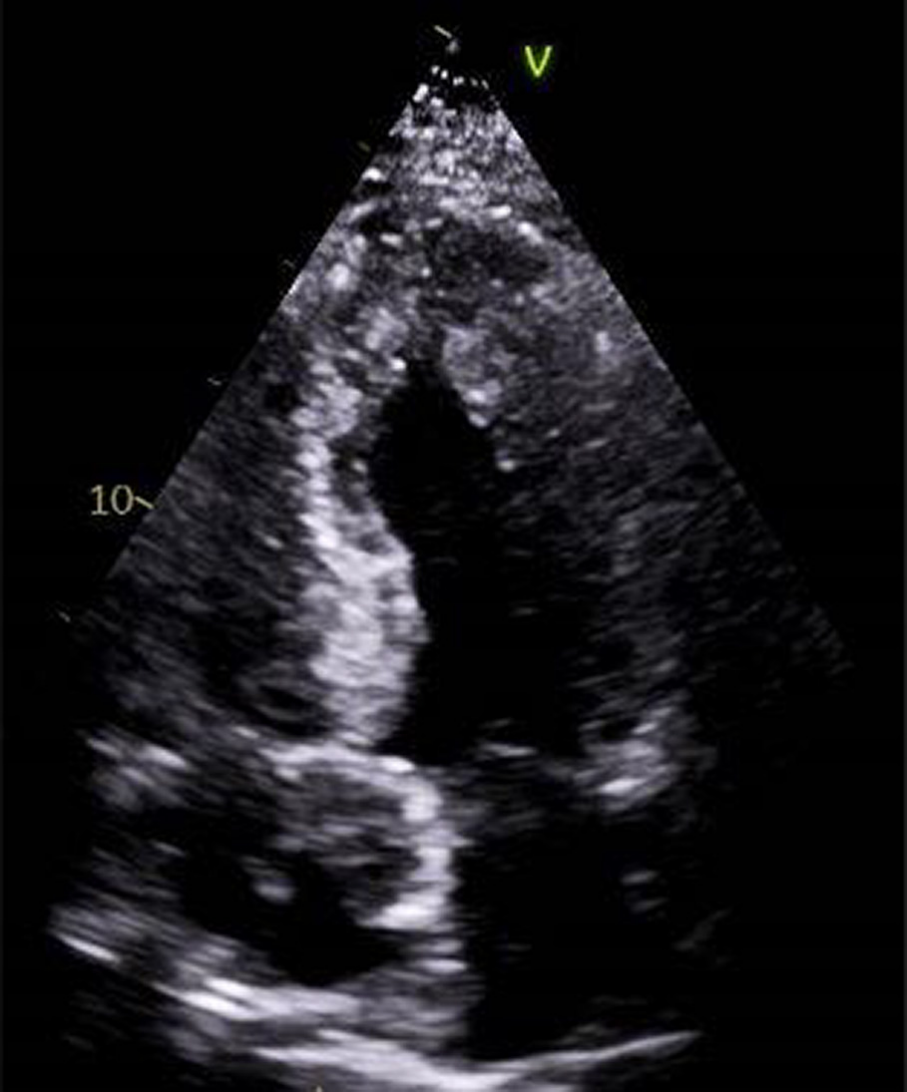
Apical 4 chamber view showing asymmetric left ventricular hypertrophy with abnormal appearance of myocardium

In September 2019, during a regular visit to the medicals for complaints of soreness in the throat, he was incidentally found to be bradycardic. He was essentially asymptomatic from this bradycardia. ECG done showed type II 2:1 AV block with premature atrial ectopics. Holter monitoring demonstrated varying degree of heart blocks including complete heart block. An echocardiogram was done which showed normal global systolic LV function (EF 56%) and no regional wall motion abnormalities. A dual‐chamber permanent pacemaker was implanted later in February 2020. Five months later, a follow‐up echocardiogram revealed moderate concentric LV hypertrophy and reduced LV systolic function (EF 42%). Abnormal hypertrophied myocardium was seen at the apex and septal wall. (Figure 1).

**FIGURE 2:**
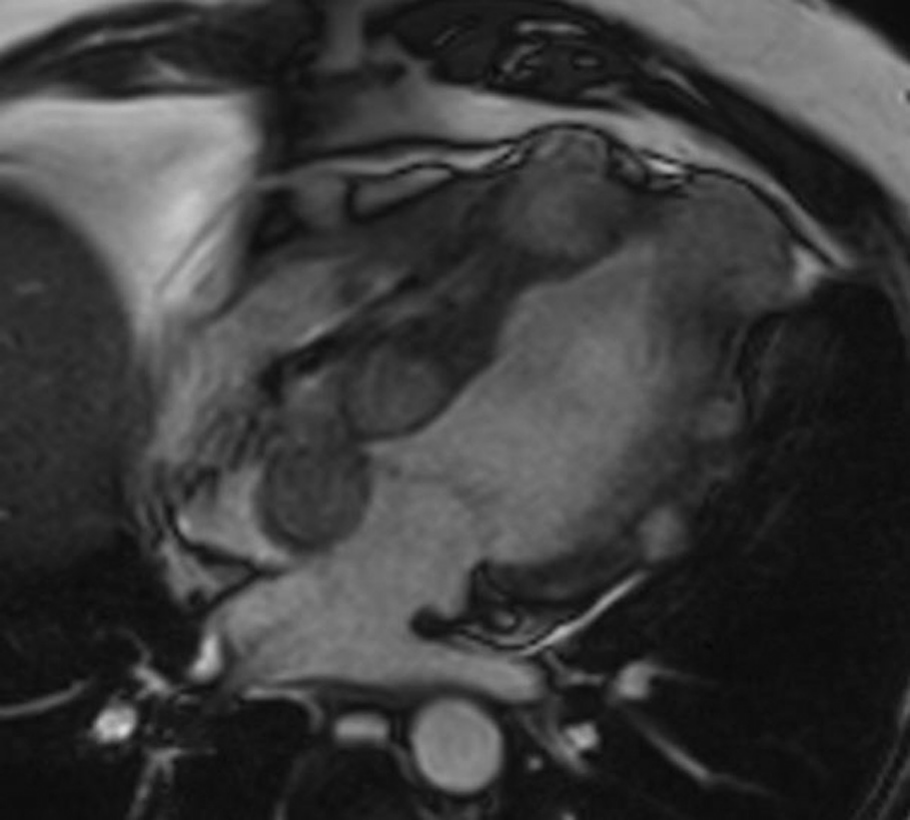
CMR, four‐chamber SSFP cine views showed multiple hypo intense masses with focal signal loss, as compared to normal myocardium extending throughout the myocardium

**FIGURE 3:**
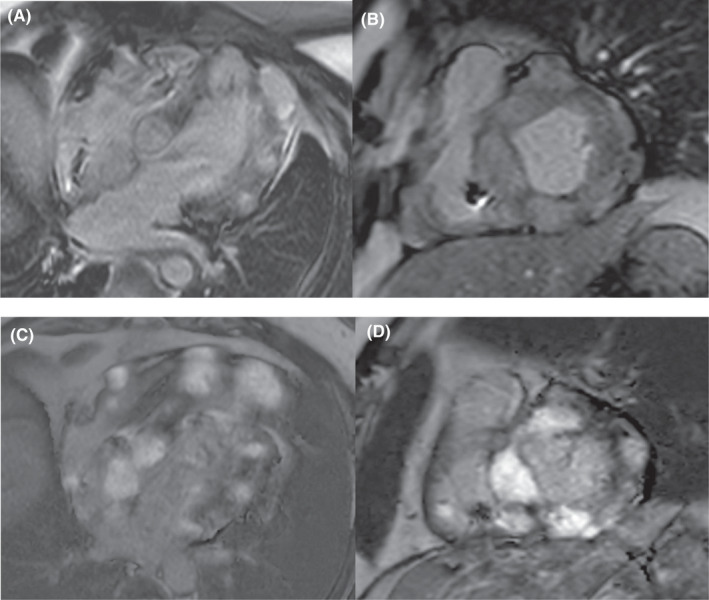
Early gadolinium (A&B) and late gadolinium (C&D) images revealed increased uptake in the multiple masses

He then underwent a cardiac magnetic resonance imaging (Cardiac MR) on a 1.5 Tesla (Philips Ingenia) scanner and findings from the multiple stacks of cine four‐chamber views confirmed the presence of hypertrophied myocardium with multiple well‐defined masses that were hypo intense with focal signal loss, as compared to normal myocardium extending throughout the left ventricle (Figure 2). T1 weighted images revealed mildly hyper intense signals. On post gadolinium, early (A&B) and late acquisition (C&D) images displayed variable degree of uptake denoting enhancement (Figure 3). Impaired left ventricular global systolic function with an EF of 45% was also seen. Cardiac MR, images acquired concluded that the masses seen signaled the presence of cardiac metastases.

**FIGURE 4:**
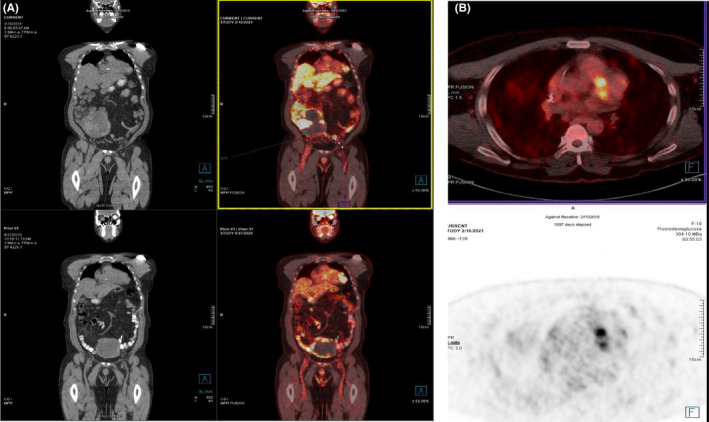
(A) PET CT coronal view showing nodules in the myocardium and mesentery along with a large abdominopelvic mass. (B) PET CT axial view showing nodules in the myocardium

Fluorodeoxyglucose (***FDG***) PET CT showed hypermetabolic nodules in the myocardium and large abdominopelvic mass. Additional large multiple hypermetabolic abdominal nodules in keeping with the large peritoneal/mesenteric deposits. Multiple small mesenteric nodules and fat stranding suspicious for malignancy. There were no metastases in the liver and lung. (Figure 4A, B).

**FIGURE 5:**
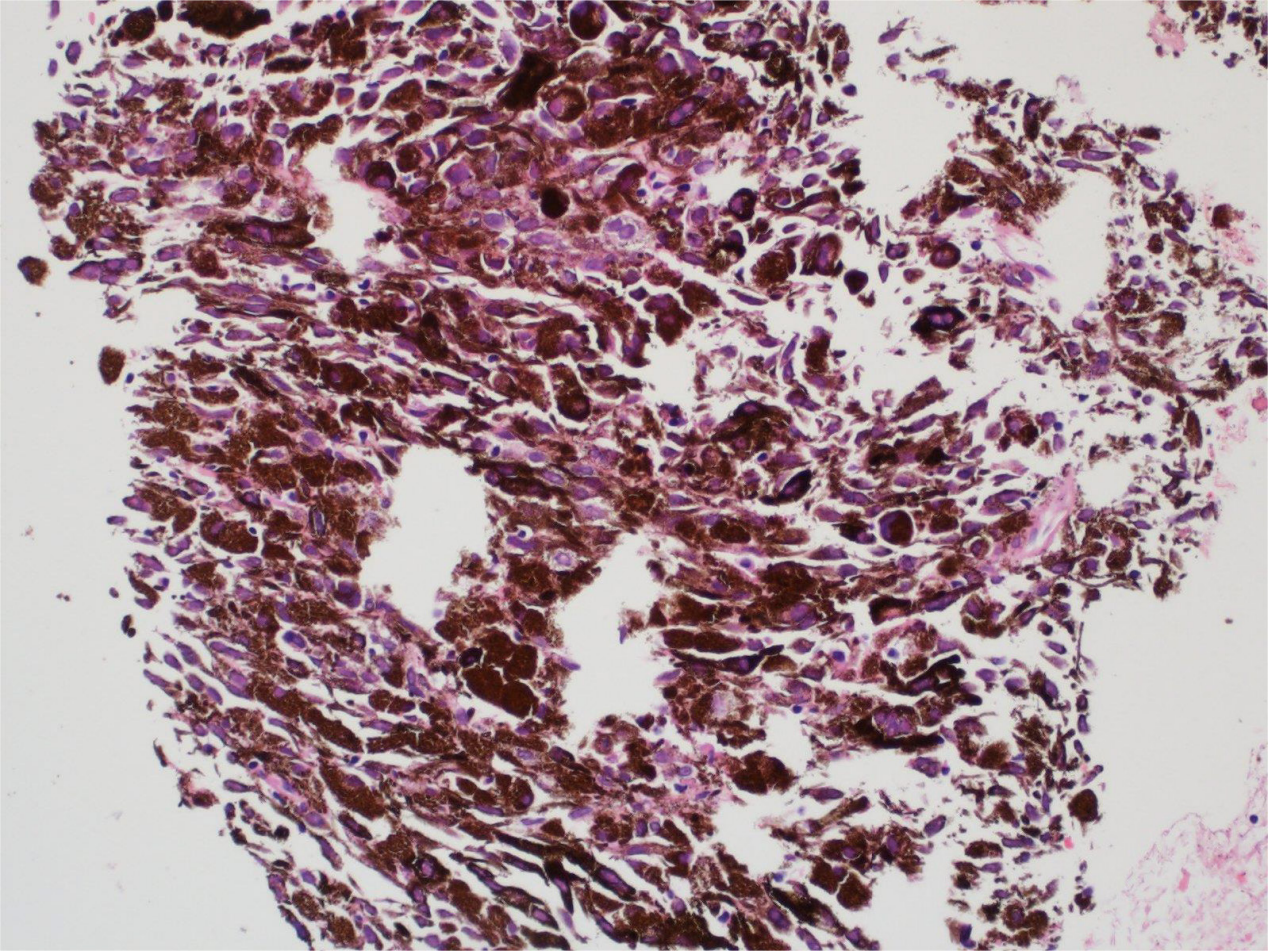
Pelvic mass biopsy revealing heavily pigmented atypical melanocytes (H and E × 20)

He underwent pelvic mass biopsy which proved to be metastatic melanoma. No mutation was detected in BRAF (Figure 5).

He was started on nivolumab 1 mg/kg and ipilimumab 3 mg/kg IV Q 3 weeks for 4 cycles followed by nivolumab maintenance for 2 cycles. He developed immune‐mediated hypothyroidism and was started on l‐thyroxine. Follow‐up PET CT revealed progression of disease in the form of newly developed bilateral lung and liver metastases and significant morpho‐metabolic progression of the abdominal deposits. He was started on second‐line nab‐paclitaxel weekly for 3 weeks and the cycle was repeated every 4 weeks.

PET CT restaging after 3 cycles showed further disease progression. Temozolomide was started as third line. PET CT scan done after 3 months demonstrated further disease progression. Temozolomide was discontinued, and currently, he is on palliative care.

## 3 DISCUSSION

In a study of 70 autopsy cases, around 50% of patients with malignant melanoma have metastases to the heart.^9^ Liver is the most frequent site of metastases from ocular melanoma.^10^ Other sites include lung, bone, skin, subcutaneous tissue, lymph nodes, kidney, thyroid, and the brain.^7^

Ocular melanoma constitutes 5% of melanomas, originates mostly from malignant transformation of uveal nevi.^11^, ^12^ Because of the lack of lymphatics in the eye, it metastasises hematogenously.^13^, ^14^, ^15^ Liver is the predominant site of uveal melanoma metastases, occurring 70%–90% of the time,^16^ compared with 15%–20% in cutaneous melanoma.^17^

With respect to cardiac metastases, early reports suggested that they are extremely uncommon,^18^ However, Makitie and Kivela suggested that although clinically the cardiac metastases from ocular melanoma are underdiagnosed, the autopsy reports indicated that 19%–24% of patients who die of disseminated ocular melanoma have cardiac metastases.^8^ Most of the patients with uveal melanoma who had metastases to the heart, also have metastases in other organs.^16^ Hematogenous route is the most common route by which the melanoma spreads to the heart.^7^

In this paper, uveal melanoma‐associated cardiac metastasis was extensively searched by expediting all the reported cases through PubMed up to September 2021, with no language restriction applied. In general, 13 reported cases were identified retrieved from nine articles,^8^, ^9^, ^18^, ^19^, ^20^, ^21^, ^22^, ^23^, ^24^ in addition to our case (Table 1). Data showed that the patients have average age of 58 (25–84) years and with equal male to female ratio.

**TABLE 1:** Reported cases of cardiac metastases secondary to uveal melanoma

Reference (Ref)	Number of cases	Age	Gender	Site of uveal melanoma	Time from enucleation to metastasis (years)	Presenting symptoms before cardiac metastasis confirmed	Modalities of conformation of cardiac metastasis	Site of cardiac metastatic involvement
Ref^8^	5	57 (47–72)	M = 3 F = 2	4 cases: choroidal 1 case: ciliochoroidal	14.7 (1.2–15.9)	All of which were Asymptomatic	Autopsy	Case 1: Left ventricular wall Case 2: Left ventricular epicardium and myocardium, and subendocardial metastasis. Case 3: The pericardium, epicardium, and myocardium in the apex of the heart Case 4: The posterior wall of the heart, together with several endo‐ and pericardial metastases Case 5: The epicardium
Ref^9^	1	43	F	Not specified	11	Pericarditis with Atrial flatter	Autopsy	Atrioventricular sulcus & subendocardial tumor nodules in the opened right atrium
Ref^18^	1	74	F	choroidal	8.9	Dizziness	Microscopic examination of excisional biopsy postmortem	Left ventricle
Ref^19^	1	84	F	choroidal	9	Near‐Syncope Attacks	Echo	left ventricular intracavitary pedunculated mass
Ref^20^	1	80	M	choroidal	15	Asymptomatic	Cardiac MRI	Left ventricle
Ref^21^	1	75	M	choroidal	15	Asymptomatic	Echo & cardiac MRI	Left ventricle
Ref^22^	1	52	F	choroidal	1	Asymptomatic	Echo & cardiac MRI	Intramyocardial masses involving both the left and right ventricles
Ref^23^	1	60	F	Not specified	NA	Asymptomatic	Echo	Left ventricle
Ref^24^	1	38	M	Not specified	13	chest pain	Biopsy	The posterior wall of the left ventricle.
Our case	1	56	M	choroidal	5	Asymptomatic	Echo, cardiac MRI & PET CT	Left and right ventricles and interatrial septum

With respect to their site of uveal melanoma, most cases have choroidal melanoma while only one case was diagnosed as ciliochoroidal melanoma.

Most of previously reported cases with cardiac metastasis of uveal melanoma were asymptomatic,^8^, ^20^, ^21^, ^22^, ^23^ while few reported cases presented with cardiac symptoms including pericarditis with atrial flatter, dizziness, near‐syncope attacks and chest pain.^9^, ^18^, ^19^, ^24^ Our patient was completely asymptomatic despite widespread metastases. The time from enucleation to confirmed cardiac metastasis was 9.2 years but in our case was 5 years only.

PET CT, MRI, and CT scan which are useful for detecting distant metastases can also detect metastasis in the heart.^25^ PET scan is frequently used for staging of melanoma and to assess response following treatment. Holder and co‐workers reported that PET had sensitivity of 94.2% and specificity of 83.3%, in contrast to 55.3% and 84.4%, respectively, for CT in the detection of metastatic sites in melanoma, especially in the liver, soft tissues, and lymph nodes.^25^ Based on our literature review, ECHO and cardiac MRI were the most common modalities of conformation of cardiac metastasis while other modalities were used, including autopsy, biopsies, and PET CT. The cardiac metastasis in our case was confirmed by Echo, cardiac MRI, and PET CT.

These cardiac metastases typically involve the pericardium and myocardium, and only rarely involve the endocardium or the valves.^8^, ^9^, ^19^, ^22^ Our patient had metastases in both the ventricles and interatrial septum most likely due to the widespread dissemination found at recurrence.

## 4 CONCLUSIONS

We recommend routine surveillance by CT scan with contrast or PET CT scan every 6–12 months after definitive treatment of uveal melanoma for a period of 10 years for medium and high‐risk patients as it may lead to earlier diagnoses of metastases resulting in more positive outcomes.

## AUTHOR CONTRIBUTIONS

AM conceived and designed the work, literature review, wrote the manuscript, overall organized the case report. NEO involved in literature review, revision of the manuscript, and proofreading the manuscript. GM provided the PET CT image. MP contributed to the Pathology section of the case report. SM and MAK contributed to the Radiology section of case report. SAK critically revised the paper and contributed to the Radiology section of the manuscript. RM managed patient care and revised the final draft of the manuscript. All authors read and approved the final manuscript and agreed to be accountable for all aspects of the work.

## CONFLICT OF INTEREST

The authors declare that they have no conflict of interest.

## ETHICAL APPROVAL

The case report was approved by the Medical Research Centre at Hamad Medical Corporation, Qatar, and the Hamad Institutional Review Board (IRB) under protocol ID MRC‐04‐21‐342.

## CONSENT

A written informed consent of patient information, images, and publication was signed by the patient before the submission of the manuscript.

## Data Availability

The datasets used and/or analysed during the current study are available from the corresponding author on reasonable request.
